# Multichannel Resonant Acoustic Rheometry System for Rapid and Efficient Quantification of Human Plasma Coagulation

**DOI:** 10.21203/rs.3.rs-3132931/v1

**Published:** 2023-07-14

**Authors:** Christina Hendren, Weiping Li, Jan P. Stegemann, Timothy L. Hall, Cheri. X. Deng

**Affiliations:** 1 Department of Biomedical Engineering, University of Michigan, Ann Arbor, Michigan, USA; 2 Department of Mechanical Engineering, University of Michigan,Ann Arbor, Michigan, USA

**Keywords:** Resonant acoustic rheometry, plasma coagulation, Coumadin monitoring, Hemostasis, Thromboelastography

## Abstract

Resonant Acoustic Rheometry (RAR), a newly developed ultrasound-based technique for non-contact characterization of soft viscoelastic materials, has shown promise for quantitative assessment of plasma coagulation by monitoring the entire dynamic process in real time. Here, we report the development of a multichannel RAR (mRAR) system for simultaneous monitoring of the coagulation of multiple small-volume plasma samples, a capability that is critical to efficiently provide improved assessment of coagulation. The mRAR system was constructed using an array of 4 custom-designed ultrasound transducers at 5.0 MHz and an electronic driving system that controlled the generation of synchronized ultrasound pulses for real time monitoring of multiple samples simultaneously. The mRAR system was tested using Coumadin-treated plasma samples with a range of International Normalized Ratio (INR) values, as well as normal pooled plasma samples. Tracking of dynamic changes in clotting of plasma samples triggered by either kaolin or tissue factor was performed for the entire duration of coagulation. The mRAR system captured distinct changes in the samples and identified parameters including clotting time, clotting speed, and the mechanical properties of the clots that were consistent with Coumadin dose and INR levels Data from this study demonstrate the feasibility of the mRAR system for the rapid, efficient, and accurate characterization of plasma coagulation.

## Introduction

Warfarin (brand names Coumadin or Jantoven), a Vitamin K antagonist (VKA), is the most frequently prescribe oral anticoagulant (OAC) for patients at risk for abnormal blood clots^1^. For example, it has been prescribed as a long-term anticoagulant therapy for prevention of stroke in patients with atrial fibrillation (AF)^2,3^, treatment of venous thromboembolism (VTE)^4^, pulmonary embolism^5^, and heart attacks^6^. Coumadin monitoring *via* regular blood testing of patients is critical to provide guidance on the proper dosage to ensure desired delayed clotting time without the risk of uncontrolled bleeding in the anticoagulation therapy^7^. Conventionally, Coumadin monitoring has relied on the prothrombin time test (PT), which reports the result as the International Normalized Ratio (INR)^8^. However, the pharmacodynamic effect of PT-monitored Coumadin (referred as PT-Coumadin) is highly variable in many patients^9,10^, making the approach less appropriate for accurate Coumadin monitoring and optimal patient care outcome.

PT measures the activity of a single clotting factor (prothrombin), which may not fully reflect the overall clotting characteristics of the blood, In contrast, viscoelasticity-based hemostatic assays (VHA), such as TEG or ROTEM^11,12^, measure the viscoelastic properties of the samples during the entire clotting process. In addition, TEG has the capability of performing multiple assays to examine the effects and interactions of multiple clotting factors with blood components. Generally, VHAs can provide comprehensive assessment of the true clotting state of the blood^13^. However, TEG and ROTEM are not widely adopted in the clinic for Coumadin monitoring due to a number of limiting factors. The equipment can be expensive and the procedure and interpretation of results require specialized training. The high cost and complexity of tests with current VHA techniques make them not feasible for point-of-care (POC) or at home tests for frequent and convenient Coumadin monitoring. Multiple assays for investigation of multiple factors require even longer time to results and further increased cost.

Resonant acoustic rheometry (RAR) is a newly developed ultrasound-based technique for non-contact, rapid characterization of viscoelastic soft materials^14^. In a recent study, we demonstrated the feasibility of RAR for quantification of coagulation characteristics of human hemophilia A plasma samples^15^. RAR utilizes a dual-mode ultrasound technique that has unique advantages for longitudinal characterization of temporally evolving soft materials in a non-contact fashion. RAR generates and detects resonant surface waves in a sample housed in a small sample holder, e.g. in a well of a standard 96-well microplate^14^, and viscoelasticity of the sample is then determined based on the measured frequency and duration of the resonant surface waves. For example, from the hydrodynamics point of view, the surface waves in liquids are capillary wave (CW) in nature, with a frequency depending on the surface tension^14,16^, $f_{CW}=\frac{1}{2\pi }\sqrt{\frac{\sigma k^{3}}{\rho },}$ where *σ* is the surface tension, *ρ* the mass density, and *k* the wavenumber of surface wave^16^. In RAR, *k* is determined by the resonant mode of the surface waves and the radius of the sample surface^14^. Thus, the surface tension can be determined from RAR measurements of the frequency of the resonant surface wave. On the other hand, Rayleigh waves (RWs) are mechanical waves that propagate on the surface of solids with a frequency^14^ $f_{RW}=\frac{1}{2\pi }\sqrt{\frac{Gk^{2}}{\rho },}$ which depends on the shear modulus of the bulk material *G*. Thus by measuring the frequency of the surface waves using RAR, the shear modulus of the material can be obtained in a non-contact fashion and as a function of time.

A single RAR measurement of the resonant surface wave in a sample takes less than 0.5 s, allowing characterization of the dynamic blood coagulation process in near real time by repeated RAR measurements throughout the entire clotting duration, e.g. a few min up to 90 min or longer. While a single channel RAR system provides rapid measurement of a single sample, it is inefficient to perform multiple samples sequentially to assess the effects of multiple factors on coagulation. The goal of this study is to develop and validate a multichannel RAR (mRAR) system for viscoelastic characterization of multiple samples simultaneously. To this end, we designed and fabricated an array of single ultrasound transducers that operate in parallel. The array is controlled by a custom-designed electronic driving system that controls the generation of synchronized ultrasound pulses with desired parameters, allowing the measurement of resonant surface waves in multiple temporally changing samples simultaneously. We tested the prototype mRAR system and demonstrated the capability of the mRAR system to quantify the coagulation of normal pooled plasma as well as Coumadin anti-coagulated plasma samples with a range of INR values.

## Materials and Methods

### Design, fabrication, and calibration of ultrasound transducers for mRAR

**Figure 1:**
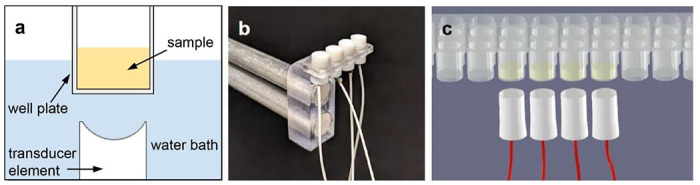
**a**) Schematics of ultrasound-based RAR measurement of a sample in a non-contact fashion. A single element transducer is placed under a sample well aiming upward towards the sample at the surface of a water tank, which provides acoustic coupling without contaminating the sample inside the well plate. The curved element focuses ultrasound from the PZT-4 crystal such that the focus of the ultrasound field is placed at the top surface of the sample (air-sample interface) for maximum signal to noise ratio (SNR) in the generation and detection of the surface movement in the sample. **b**) Photo of an array of 4 custom-designed and fabricated ultrasound transducer elements in a mounting piece. Wires from the elements are connect to the electrical driver (not shown). The transducer assembly is connected to a motion controller (not shown) for 3D alignment for RAR measurements. **c**) Schematic showing an array of 4 transducers aligned and placed below a set of 4 samples in a 96-well microplate for simultaneous measurements.

To develop an mRAR system for measuring multiple samples housed in a standard 96-well microplate, computational simulation was performed before fabrication and testing. COMSOL simulation determined that the 3-dB width of a focused ultrasound beam should be ~ 0.5 mm at the focus in order to generate resonant surface waves in a sample within a well of radius of 3.25 mm in a standard 96-well microplate, as previously described^14^ (Fig. 1A). Simulation was also performed using the MATLAB K-WAVE package that determined the focal distance of a 5.0 MHz transducer (with an active surface diameter 5.0 mm) to be 12.3 mm for the desired mRAR system.

Rapid prototyping was employed to fabricate single element transducers ^17^ using a 5.0 MHz piezoelectric ceramic crystal (PZT-4, Steiner & Martins, Inc.) and a plastic elliptical lens for each transducer to achieve the geometric focus of 12.3 mm. The acoustic lens and housing of the transducer were 3D-printed (Stratasys J750 printer, opaque material with an acoustic impedance of ~3.1 MRayl) from CAD designs made on Autodesk Inventor^17^. The 2-piece design allowed placement of the PZT crystal inside of the housing and secured with epoxy as an adhesive, backfill, and waterproofing sealant that protected the wiring within the housing cylindrical chamber (Epotek E-120HP and E-00NS, Loctite, Dusseldorf, Germany). This modular design permitted fabrication and testing of individual transducers separately before assembly into the final array.

To demonstrate the general principle of mRAR, a prototype mRAR transducer assembly was constructed including 4 single transducers with the same design parameters. These single transducers were mounted on a 3D-printed supporting scaffold that holds the transducers (Fig. 1B) with a well-to-well distance from each other that physically aligned them to 4 adjacent wells in a 96-well microplate (9 mm center-to-center spacing) (Greiner Bio-One), allowing for testing of 4 samples simultaneously (Fig. 1C).

Calibration of the transducers was performed using a fiber optic hydrophone (Onda HFO 690) by measuring the acoustic pressure field of the transducers in de-gassed, room temperature water in a free field condition. The hydrophone was operated at a sensitivity of 7.00 mV/MPa or higher and was spatially scanned at a step size of 0.1 mm to measure 3D field maps of acoustic pressure distribution.

### Electrical circuit for mRAR operation

An electric driving circuit was designed and constructed to operate the array of transducers to function in both transmit-only and transmit-receive (or pulse-echo) mode in a synchronized fashion for mRAR measurement. A class-D driver circuit architecture used for early histotripsy research ^18^ was modified to use Silicon Carbide transistors for operation at 5.0 MHz. This design uses a pair of transistors operating in a “push-pull” mode to generate a high voltage square wave burst which is then filtered by a series inductor to produce a higher voltage, nearly sinusoidal output for driving the transducers (**Fig. S1**). Varying the main power supply voltage to the transistors varies the amplitude of the square wave and ultimately the transmit pulse amplitude. For these experiments, the maximum square wave was 75 V, producing a driving sinusoid of about 190 V peak-peak. To receive acoustic echoes from the surface of the sample, a 50 Ohm current sensing resistor was placed in series in the ground return path from each transducer. The voltage on this resistor was digitized by a model 5443 Picoscope (Pico Technology, St Neots, UK). On transmit, this resistor is bypassed by a pair of transistors to maximize power output and prevent saturating the digitizer (**Fig. S1**).

A custom microcontroller implemented on a field programmable gate array (FPGA) interfaced the circuit board to a host computer with software control through MATLAB. The electrical circuitry was designed to generate ultrasound pulses with controllable acoustic pressure amplitude, number of cycles (pulse duration), and pulse repetition frequency of the ultrasound pulses such that the same transducer served for both pushing and detection in RAR, as described previously where two separate transducers and driving systems were used for these different tasks^14^.

For RAR measurement, the transducers were driven to generate an ultrasound tone burst in transmit mode (excitation pulse) to induce surface perturbation leading to resonant surface waves in the sample. After generation of the tone burst, the transducers were immediately switched electronically to pulse-echo mode, first generating a short pulse (detection pulse) and then receiving its backscattered signal (*via* the Picoscope) from the sample surface at a high pulse repetition frequency (PRF) (e.g. 10 kHz). Thus the resonant surface wave in the samples was measured in real time by detecting the temporal shift of the echoes relative to the equilibrium surface to monitor the changes in the samples.

### Sample preparation and workflow of mRAR measurements of clotting plasma

Pooled human plasma and individual subject (Coumadin) plasma were purchased from George King Bio-Medical, Inc. (Overland Park, Kansas) with International normalized ratio (INR) values in the range of 1.5-2.8, 2.9-4, and 4+ (as assessed by George King Bio-Medical Inc.) representing low, medium, and high dosage of the anti-coagulants. Plasma samples were grouped based on the INR values. Low, medium, and high INR groups included plasma samples with INR of 1.5 - 2.8, 2.9 – 4.0, above 4.0, respectively.

The mRAR system was tested for characterizing the effects of Coumadin on coagulation of plasma induced by tissue factor (TF) and Kaolin respectively. To initiate clotting of the plasma samples, clotting reagents CaCl_2_ (final concentration 13 mM) (Fluka) and Rabbit Brain Cephalin (25 μM) (Pel-Freez Biologicals) were added, in addition to 1:500 dilution of TF (Innovin, Siemens) or 2% w/w of kaolin (Haemonetics). Tris buffered saline (Fisher) was used in dilution of 1X and bovine serum albumin (Fisher) added at 0.1%. A single clotting reagent solution was formed by combining the needed reagents for a coagulation assay. For each sample under a given clotting condition, 4 replicates were measured simultaneously using the mRAR system.

Plasma samples and clotting agents were warmed to 37 °C before mRAR experiments. The desired clotting reagent (volume 10 μl per well) was mixed with the plasma sample (90 μl per well) and transferred as quickly as possible to 4 adjacent wells in a 96-well microplate just before RAR experiments. The duration for the process of mixing and sample transferring was 87.0 s ± 16.0 s for all experiments. A tank of degassed water at 37 °C was used to provide acoustic coupling for the transducers and the microplate with only its bottom submerged in water to maintain sterile condition for the samples. The transducers aimed upward to 4 individual wells housing the samples. A computer-controlled 3D motorized positioner (Velmex) controlled the position of the transducers, aligning each focus at the center of the top surface of the respective sample in a well. The focus of the transducer in the vertical direction was confirmed by the time of travel of the pulse to and back from the top surface of the sample (at sound speed of 1480 m/s).

### Signal processing to determine the surface displacement from RAR measurements

As described previously^14^, a custom-developed MATLAB script was used to analyze the measured backscattered signals during pulse-echo detection during RAR to determine the surface displacement in a sample after application of each “push” ultrasound pulse. The temporal change of the surface displacement was obtained as a function of a fast time, *τ*, which was defined as the oscillation time for the resonant surface wave after each surface perturbation initiated by a push ultrasound pulse. Briefly, after the application of a “push” pulse at a given observation time *T*, a series of N pulse-echo operations were applied sequentially at T+τ=T+nΔτ with an interval of Δτ, where n = 0 to N and *τ* is defined as the fast time. The time interval is the inverse of the pulse repetition frequency (PRF) of the pulse-echo operation. For each pulse-echo application at a given fast time *τ*, the surface displacement was determined based on the change of the traveling time *t* of the echo signals from the sample surface relative to that from the surface at equilibrium determined by cross-correlation of the signals assuming a constant and uniform speed of sound. At a constant sound speed, the changes in the time of travel of the ultrasound pulse to and from the sample surface is proportional to the surface displacement. The cross-correlation analysis used the averaged echo signals preceding the push signals to mitigate variation of pulse amplitude. The surface displacement as a function of oscillation time, S(T+τ)*,* was then determined from all the echo signals. The corresponding power spectrum of S(T+τ) with regard to fast time was obtained by performing a Fast Fourier Transform (FFT)^14^.

To examine the changes in the surface movement (resonant surface wave) in the samples during coagulation over observation time *T*, repeated RAR applications with an interval of ΔT = 6.0 *s* were used to obtain the surface displacement as a 2D matrix $S\left(T_{m},\tau_{n}\right)$ *,* where m and n depicted the observation time point and the surface oscillation time. A 2D heatmap of $S\left(T_{m},\tau_{n}\right)$ was generated (vertical axis: fast time *τ*; horizontal axis: observation time). Correspondingly, a spectrogram was generated by displaying the power spectra of $S\left(T_{m},\tau_{n}\right)$ during coagulation. RAR measurement data was collected on each sample for a total of 60 minutes.

### Determination of coagulation parameters and statistical analysis

The frequency of the resonant surface wave was identified as the peak in the power spectrum of the surface displacement measured in RAR. A spectrogram of the frequency is generated to show the changing power spectrum as a function of coagulation time, and was used to extract a set of parameters for the quantification of plasma coagulation^15^. The initial frequency, *f_int_*, was calculated as the averaged frequency measured during the first minute of RAR measurements of a sample while the final frequency, *f*_*end*_, as the averaged frequency measured during the last 5 minutes of RAR measurements. The clotting start time, *T*_*int*_, was defined as the time at which the resonant frequency began to increase by 5% from *f*_*int*_ determined from the spectrogram. The clotting end time, *T*_*end*_, was defined as the time at which the frequency reached 95% of *f*_*end*_ , indicating the end of the active clotting process. The clotting duration was the difference between these times, *T*_*end*_ – *T*_*int*_, A visual representation of these parameters is shown in **Fig. S2**.

The results obtained from coagulation of pooled normal plasma as well as plasma samples with low, medium, and high INR triggered by either TF or Kaolin were compared. Linear regression with interaction effects was performed. Differences among the groups were determined to be statistically significant if p < 0.05.

## Results

### Streamlined mRAR system operation based on dual-mode operation of array of single transducers

**Figure 2.**
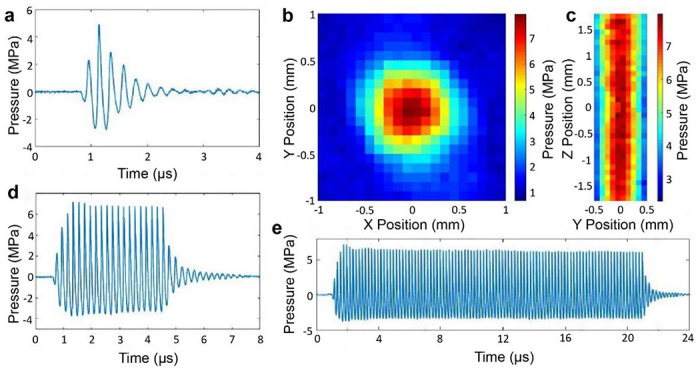
Characterization of the ultrasound field of the mRAR system using a fiber optic hydrophone. **a**) A typical example of a 2-cyle pulse (for imaging and detection) generated by one of the transducers. The output across the 4 elements had averaged pressure amplitude of 3.4 ± 0.8 MPa. **b&c**) The acoustic pressure field maps of the transverse focal plane and axial distribution measured using a 2-cycle pulse with a 0.1 mm step size, with the averaged full width half maximum (FWHM) of 1.02 ± 0.07 mm in the X direction, 1.02 ± 0.06 mm in the Y direction, and 12.41 ± 4.11 mm in the Z direction respectively. **d&e**) Example of tone bursts with 20 cycles or 100 cycles respectively used for excitation of surface perturbation in the sample in RAR. The averaged pressure amplitude for 20-cycle pulses was 4.6 ± 1.1 MPa

As shown in Fig. 1, in the array driven by the control circuit, each of the transducers produced short ultrasound pulses (Fig. 2a) for pulse-echo mode operation with a pulse duration of 0.6 μs, attaining an acoustic pressure amplitude of 3.4 ± 0.8 MPa (*n* = 4) at a 75.0 V input setting. The transducers generated symmetric focused ultrasound fields, as shown by the spatial distribution of the acoustic pressure in the focal plane (Fig. 2b). The full width half maximum (FWHM) of the focal zone was measured to be 1.02 ± 0.07 mm and 1.02 ± 0.06 mm in the in the X and Y direction respectively (Fig. 2b), satisfactorily meeting the design requirement for the mRAR system. The FWHM in the Z (axial) direction was 12.41 ± 4.11 mm, showing an elongated focal depth (Fig. 2c), providing flexibility for axial placement of the focus relative to the sample surface during RAR measurements. The transducers were also able to generate tone bursts with an adjustable number of cycles to serve as the excitation or pushing pulse to induce surface waves in the samples, as shown by the examples of tone burst with 20 cycles and 100 cycles respectively (Fig. 2d&e). As expected, tone bursts with longer duration achieved slightly higher acoustic pressure amplitudes than the 2-cycle pulses for pulse-echo operation (imaging mode). The spatial-peak temporal-peak intensity (I_sptp_) for a 2-cycle pulse (measured across the four transducers) was 484.3 ± 187.5 W/cm^2^. The I_sptp_ was 548.6 ± 192.9 W/cm^2^ and 640.0 ± 311.2 W/cm^2^ for a 20-cycle and 50-cycle pulse respectively.

**Figure 3.**
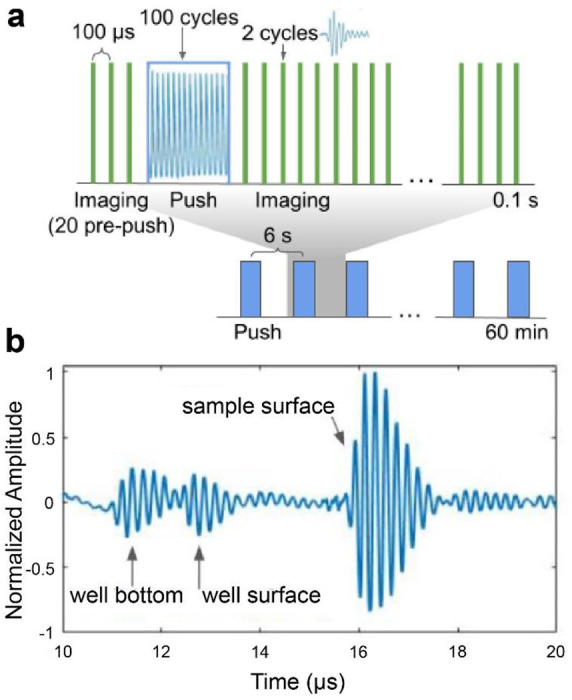
Synchronization of the excitation tone burst and the detection pulses in RAR. **a**) The pulse sequence for one RAR measurement shows that 20 imaging/detection pulses are applied at a pulse repetition frequency (PRF) of 10 kHz to obtain the baseline of the equilibrium position of the sample surface. Then a single ‘push’ pulse (tone burst) consisting of 100 cycles (shown within the blue box) is applied to induce a surface wave, followed by repeated applications of imaging pulses repeating every 100 μs (PRF 10 kHz) for 0.1 s to measure surface movement. **b**) A representative backscattered signal received by a transducer, showing the echo signals from the two surfaces of the well plate as well as the echo from the top surface of the sample (air-sample interface). The time difference of these echoes reflect the thickness of the plate bottom and the spatial distance of the sample surface assuming constant sound velocity. The reflected signals received from the repeated pulse-echo detection within the 0.1 s window are used to determine the temporal shift of the echoes using cross-correlation to obtain the surface displacement.

The mRAR system successfully produced a set of synchronized pulses for both excitation and detection of surface waves in 4 samples simultaneously for RAR measurement. As illustrated in Fig. 3a, for each RAR measurement at a given time, 20 imaging/detection pulses (PRF from 5 to 10 KHz) were applied to obtain the base-line signals from the surface of the samples at equilibrium before application of a single tone burst or a ‘push’ pulse (duration 20 μs) to induce surface resonant wave in the samples. The surface movement was measured using 500 detection pulses immediately after the “push” pulse to detect the temporal shift of the echo signals from the sample surface relative to the equilibrium. The example in Fig. 3b shows the A-line signal measured by one of the transducers operated in pulse-echo mode, showing the reflective signals from different interfaces including the sample surface.

### RAR detected dynamic changes during plasma coagulation in real time

**Figure 4.**
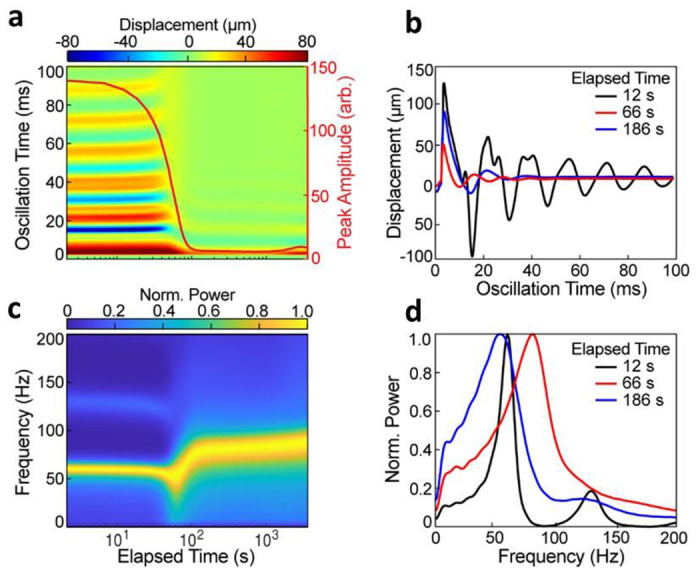
An example of RAR measurement showing coagulation of normal plasma triggered by TF. **a**) Color-coded heatmap of the dynamic surface displacement. The vertical axis is the oscillation time indicating how the surface is oscillating, while the horizontal axis shows the elapsed time when the RAR measurement was performed every 6.0 s for 60 min. The heatmap as well as the red line plot clearly show a significant and rapid decrease in the surface displacement amplitude and duration at 90 s, indicating liquid-to-solid phase transition corresponding to clotting of the sample. **b**) Selected examples of the surface displacement plots before, during, and after the rapid clotting phase, showing the changes in amplitude and duration of the surface displacement with the characteristics of damped harmonic oscillator. **c**) Spectrogram of the surface displacement in **a**) shows that the significant increase in frequency occurred at the same time, at 90 s, at the liquid-to-solid phase transition. **d**) Corresponding power spectra of those in **b**) show a general trend of increasing and widening of the frequency peak of the surface wave.

To test the performance of the mRAR system, we conducted experiments to measure the changes of plasma samples during coagulation. The results in Fig. 4 show a representative example of a series of RAR measurements (measurement interval of 6.0 s for a duration of 60.0) of coagulation of normal plasma sample triggered by TF. The heat map of the surface displacement (Fig. 4a) displays the surface displacement at the center of the sample surface as a function of coagulation (elapsed) time, revealing significant changes in the duration and amplitude of the surface waves in the sample during the coagulation process (Fig. 4a&b). Clearly, the resonant surface waves or surface movement generated in the samples exhibited the characteristics of a damped harmonic oscillator (Fig. 4b). Overall, the RAR measurements reveal several distinct stages during plasma coagulation. During the initial phase of the coagulation, i.e. T < 90.0 s, the amplitude and period of the surface displacement remained relatively constant (Fig. 4a), suggesting a lag phase of coagulation during which no changes in the mechanical properties occurred in the sample. However, during the period of *T* = 90.0 s – 150.0 s after the lag phase, the amplitude and duration of the surface waves significantly reduced, indicating high damping (Fig. 4a), suggesting a liquid to solid phase transition and active phase of coagulation in the plasma sample. Lastly, when T > 150.0 s, the resonant surface wave in the sample stabilized in terms of both amplitude and duration, indicating the sample has reached the completion of the coagulation (Fig. 4a&b).

The corresponding spectrogram (Fig. 4c), which is the normalized power spectrum of the surface displacement/wave vs. coagulation time, also provided a global view of the changes occurring in the samples during coagulation. It revealed several stages throughout the coagulation process (Fig. 4c&d). During the lag phase, the frequency of the resonant surface waves remained constant (Fig. 4c), suggesting a liquid state of the samples. After the lag phase, the frequency of the resonant surface wave started to undergo a period of rapid increase (Fig. 4), suggesting the emergence of elasticity in the sample as it transitioned from liquid to solid, with the surface wave transitioning from CWs to Rayleigh behavior^14,16^. The frequency peak widened concurrently with the rapid increase of frequency (Fig. 4c&d), suggesting increased viscosity of the sample due to clotting^16^. Lastly, when T > 150.0 s, the surface wave frequency stabilized (Fig. 4c), reflecting completion of the clotting process and formation of a stable clot with stabilized shear modulus *G*.

Taken together, these results demonstrate the capability of our mRAR system for capturing the dynamic progression of plasma coagulation. The drastic decreases in resonant wave amplitude and duration, as well as the increase in the frequency of the resonant surface waves, provide a clear and sensitive indicator of clot formation dynamics as the sample transitioned from fluid to solid.

### Characterization of Coumadin effects on plasma coagulation using mRAR

**Figure 5.**
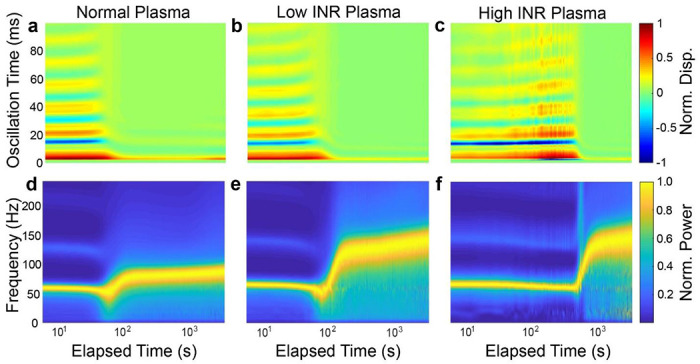
Representative results of RAR measurements of plasma samples during clotting triggered by Tissue Factor (TF). **a-c**) Heatmaps of the surface displacement in normal, low, and high INR samples, respectively. Delayed clotting is detected in plasma samples with increasing INR values, as evidenced by the significant decrease in the amplitude of the surface displacement due to the liquid-to-solid phase transition in the samples. **d-f**) The corresponding spectrograms of those in **a-c** show the rapid increase in the frequency of surface waves corresponding to the decrease in amplitude of the surface waves shown in the heatmaps.

**Figure 6.**
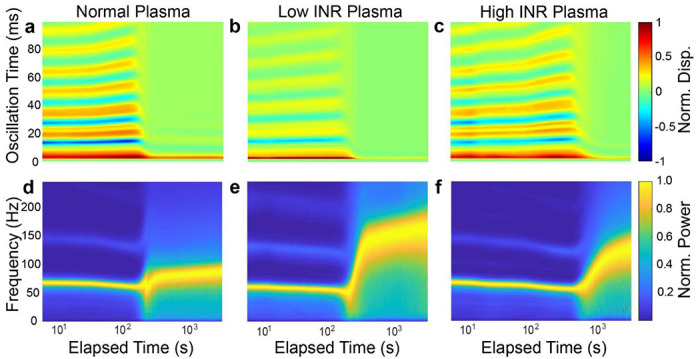
Representative results of RAR measurements of plasma samples during clotting triggered by Kaolin. **a-c**) Heatmaps of the surface displacement in normal, low, and high INR samples, respectively. Delayed clotting is detected in plasma samples with increasing INR values, as evidenced by the significant decrease in the amplitude of the surface displacement due to the liquid-to-solid phase transition in the samples. **d-f**) The spectrograms of those in **a-c** show the rapid increase in the frequency of surface waves corresponding to the decrease in amplitude of the surface waves shown in the heatmaps.

Experiments were performed using the mRAR system to characterize the effects of Coumadin on the coagulation of human plasma samples induced by Tissue factor (TF) or Kaolin, respectively, as described in Ma terials and Methods. As shown by the example in Fig. 5, heatmaps of surface displacement and corresponding spectrograms revealed clear differences between groups in terms of the dynamic progression of the samples during TF-triggered (Fig. 5) or Kaolin-triggered (Fig. 6) coagulation. While the heat maps and spectrograms showed similar characteristics in general, delayed coagulation was clearly evident in Coumadin samples with increasing INR, as reflected by the rapid decreases in surface movement amplitude/duration and concurrent increase in frequency. The examples in Fig. 5 & 6 also show differences of coagulation dynamics triggered TF and Kaolin.

**Figure 7.**
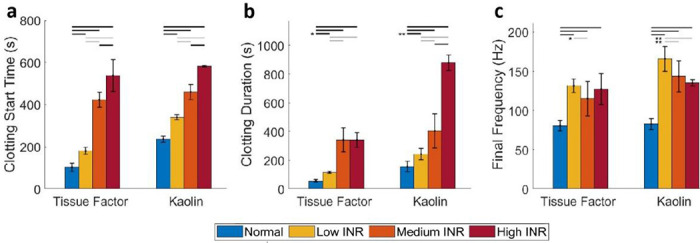
Comparison of the RAR parameters of coagulation of plasma samples. **a**) Clotting start time, or the time when phase change occurs, extracted from the spectrograms from different groups of plasmas (normal and Coumadin patient plasma at dosages of low, medium, and high INR) triggered by TF (n= 9, 8, 8,10) or Kaolin (n= 11, 7, 8, 4) respectively. **b**) Clotting duration, determined as the period of time from the start to end of the rapid coagulation phase for normal and Coumadin plasmas with different INR values. **c**) Final frequency measured at the end of coagulation at 60 min for normal and Coumadin plasmas with different INR values. The differences between groups are statistically significant, as indicated by horizontal bars when p < 0.001, * when p < 0.05, or ** when p < 0.01.

We performed quantitative assessments of the coagulation dynamics using parameters derived from the measured frequency of the resonant surface waves (**Fig. S2**), and the results are shown in Fig. 7. Overall, RAR detected statistically significant differences in coagulation between groups triggered by TF or Kaolin (**Tables S1-S3**). In general, the parameters exhibited similar trends as INR values, although there were several distinctions. As shown by the results in Fig. 7a, plasma samples with higher INR values had a delayed Clotting Start Time compared to normal plasma samples initiated by both TF and Kaolin. TF appeared to initiate a faster Clotting Start Time in normal and lower INR plasma samples compared to Kaolin, while there are no differences in Clotting Start Time due to TF or Kaolin for medium and high INR samples (Fig. 7a), potentially capturing the effects of different coagulation pathways. In terms of clotting duration, the medium and high INR samples took longer to coagulate than the normal and low INR samples triggered by TF (Fig. 7b). However, the high INR samples exhibited significantly increased clotting duration triggered by Kaolin compared to samples in other groups, suggesting that clotting duration is especially sensitive to high INR values when triggered with Kaolin (Fig. 7b). Interestingly, the final frequency of the resonant surface waves in normal plasma samples measured at the end of coagulation was significantly lower than those measured in plasmas samples from patients treated with Coumadin (Fig. 7c). Since the frequency reflects the stiffness of the final clots, these results suggest that normal plasma samples formed softer clots than plasmas with Coumadin, providing potentially useful information regarding the effects of Coumadin. While no clear trend was present in terms of the final frequency, or clot stiffness vs. INR values in the TF-triggered experiments, kaolin-triggered coagulation showed that plasma with the lowest INR values had a higher final frequency compared to plasma with higher INR values (Fig. 7c). The frequency results of Kaolin-triggered coagulation exhibited a trend opposite to the clotting duration vs. INR values (Fig. 7b), indicating that higher INR values delayed coagulation and formed softer clots, although still much stiffer than normal clots.

## Discussion

A previous RAR system^14^ used two co-linearly aligned transducers with different center frequencies and characteristics for generating either a tone burst excitation pulse or short imaging pulses for pulse-echo detection. While such a dual transducer system may optimize excitation (at lower frequency of 1.5 MHz) and detection (at higher frequency of 7.0 MHz), it requires two separate electronic systems with different functionalities that need to synchronize and spatially align the ultrasound pulses, and is therefore not well-suited for the construction of multichannel systems due to increased cost and complexity. While the throughput of such a system can hypothetically be increased by multiplexing and automatically translating the transducer assembly to different samples, these functionalities are prohibitive to constructing a multi-channel RAR system for simultaneous RAR measurements of multiple samples in systems with fast-changing properties such as hypercoagulation. In contrast, the mRAR system described here employed the same ultrasound transducer for both excitation and detection with an innovative design for a streamlined electronic driving system that achieved both transmit and transmit-receive modes with controllable pulse duration and pulse repetition frequency.

The multi-channel RAR system has the general advantages of rapid testing of plasma coagulation objectively, rapidly, and efficiently. The system is cost-efficient and can be easily constructed. It allows for testing of sample replicates under identical conditions, or for testing sample from the same patients using multiple assays simultaneously. Importantly, mRAR is a VHA-based technique that provides quantitative information on sample properties over the entire clotting process in a cost-efficient fashion. Therefore, mRAR offers a promising approach as a point-of-care (POC) device for more frequent and convenient testing for patients at home or in the clinic for personalized health care^19,20^. The technique overcomes the limitations of both conventional PT and VHA techniques such as TEG or ROTEM^11,12^. A criticism of clinically-available VHA methodologies is that they considerably deform the clot sample, as they use either a rotating cup and pin system (e.g., TEG 5000, ROTEM delta, and ROTEM sigma), vertical cup and pin system (e.g., Sonoclot), or vibration detection by light emitting diode (e.g., TEG 6s)^21–23^. New and emerging technologies of point-of-care (POC) hemostatic assays have used iterations of ultrasonic deformation methodology^12^. For example, sonic estimation of elasticity via resonance (SEER) is a rheological method that addresses this drawback of contact-based techniques by utilizing ultrasound technology. Quantra^®^ is a fully-automated, ultrasound-based test that uses dry reagents, thus simplifying quality assurance and improving reproducibility^12,24,25^. However, these VHAs require expensive systems and reagents that are not readily available at every institution that could benefit from these devices.

RAR technology is based on an entirely different operating principle from other ultrasound-based approaches^26^. It specifically measures the surface waves and extracts the viscoelasticity based on the dispersion relation of the surface waves in different mechanical regimes. It therefore provides a unique way to measure viscoelastic properties including surface tension, viscosity, and shear modulus all at once^14^. While a 4-channel mRAR system was developed in this study, the mRAR platform is easily expandable to more channels. Since RAR measurement requires less than 0.5 s and clotting is much slower, an alternative strategy would be to use a motion controller to scan the array of 4-transducers to measure additional sets of samples in a multiplexed fashion to improve throughput.

The clotting times measured in this study using mRAR are in the same range as those found in a previous study using ROTEM^®^^13^, which also supported our results showing the absence of trends between TF-triggered clotting and clotting duration and final frequency, even though increasing concentrations of contact activator accelerated clot formation. This effect was hypothesized to be related to TF’s role in triggering thrombin formation within the coagulation cascade. With an excess of TF, an intrinsic complex (FIXaFVIIIa) could be bypassed, resulting in slower clotting. Alternatively, an excess of TF may have caused saturation of factor VIIa, resulting in a decline in maximum velocity (MaxVel on ROTEM^®^) observed for platelet poor plasma, platelet rich plasma, and whole blood^13^. Further testing of mRAR for characterization of clotting dynamics using whole blood is needed to more fully validate the mRAR approach.

Coumadin is the most frequently prescribed oral anticoagulant to reduce the risk of blood clots. Accurate Coumadin monitoring is critically important to determine the proper dosage of the anticoagulation therapy to ensure efficacy and safety. Conventional Coumadin monitoring uses prothrombin time (PT) of patient plasma and the associated international normalized ratio (INR), but with high patient variability. Similar to characterization of coagulation of human hemophilia plasma samples^15^, this study used the final frequency of the resonant surface waves as a convenient way to assess Coumadin effects and clotting agents. Nevertheless, the measured surface displacement amplitude and frequency could be used to determine viscosity and shear modulus^14^, which may be useful for more comprehensive studies in the future. Other potential factors affecting the accuracy and consistency of RAR measurements include the misalignment of the ultrasound pulses at the center of the sample surface and the variation in the time it took to prepare the samples at the beginning of the experiments. While our prototype mRAR system uses a water tank as an easy approach for acoustic coupling while maintaining the sterile condition for the samples, effort is underway to develop ultrasound transducer system without the use of water tank for easy transportability.

## Conclusion

The results of this study show that a prototype mRAR platform provided streamlined operation for real-time monitoring of the coagulation process in multiple plasma samples simultaneously. The new mRAR system reduced the design and operational complexity of our previously reported RAR technology. Characterization of the mRAR system confirmed that the system met design criteria in generating desired ultrasound pulses for RAR measurements during coagulation of human plasma samples. Results demonstrated that the mRAR system was capable of efficiently measuring 4 plasma samples simultaneously during coagulation. The mRAR measurements clearly identified different clotting characteristics between normal and Coumadin-treated plasma samples triggered by TF or Kaolin, revealing the sensitivity of mRAR parameters to different conditions.

## Data Availability

Data included in this manuscript is available and will be shared upon request. Contact Cheri Deng: cxdeng@umich.edu.
